# Cryptococcal Meningitis in an HIV-Negative, Lymphoma Survivor: A Case Report of an Uncommon Dual Pathology

**DOI:** 10.7759/cureus.97205

**Published:** 2025-11-19

**Authors:** Momna Arif, Sufian Jawed, Anum Kashif, Asghar Alishah, Mujeeb Ullah Makki

**Affiliations:** 1 Department of Acute Internal Medicine, Midland Metropolitan University Hospital, Birmingham, GBR; 2 Department of Geriatric Medicine and Stroke, University Hospitals Birmingham National Health Service (NHS) Trust, Birmingham, GBR; 3 Department of Internal Medicine, Central Park Teaching Hospital, Lahore, PAK; 4 Department of Gastroenterology, Midland Metropolitan University Hospital, Birmingham, GBR

**Keywords:** abducent nerve palsy, cardiomyopathy, crytococcal meningitis, hiv negative, lymphoma survivor, stroke

## Abstract

Cryptococcal meningitis, though uncommon, can present in individuals with compromised immunity, including those without HIV but with a history of immunosuppressive treatments. We describe the case of a 74-year-old woman with a prior diagnosis of non-Hodgkin lymphoma in remission who developed cryptococcal meningitis following a recent right insular cerebral infarct. She also exhibited a left abducent nerve palsy, raising concern for elevated intracranial pressure. Diagnostic workup revealed a cerebrospinal fluid cryptococcal antigen titre of 1:2650 and positive polymerase chain reaction (PCR) for *Cryptococcus neoformans*, confirming the infection. Her clinical course was further complicated by anthracycline-induced cardiomyopathy and pulmonary embolism. The patient was treated with liposomal amphotericin B and fluconazole, followed by the addition of flucytosine, which led to microbiological clearance, resolution of the cranial nerve palsy, and overall clinical improvement. This case highlights the need for timely cerebrospinal fluid (CSF) analysis and a multidisciplinary approach when evaluating unexplained neurological symptoms in patients with prior oncologic treatment.

## Introduction

An opportunistic infection, cryptococcal meningitis is a severe fungal infection of the central nervous system, classically seen in immunocompromised individuals, particularly those with advanced HIV infection [1], accounting for an estimated 15% of AIDS-related deaths worldwide. Cryptococcal meningitis is increasingly being recognized among HIV-negative patients with acquired immunosuppression, such as those undergoing chemotherapy, especially for haematologic malignancies or receiving long-term steroid therapy [1,2]. Cryptococcal meningitis is diagnosed through cerebrospinal fluid analysis, fungal detection (India ink, culture, or antigen tests) and brain imaging. Treatment involves a three-phase antifungal regimen: induction with amphotericin B plus flucytosine, consolidation with high-dose fluconazole, and long-term maintenance with lower-dose fluconazole to prevent relapse. Recent cerebrovascular events may further predispose patients to opportunistic infections by compromising cerebral perfusion, microglial surveillance, and integrity of the blood-brain barrier [3]. Here, we present a rare case of cryptococcal meningitis arising shortly after an insular stroke in an HIV-negative, post-chemotherapy patient, complicated by sixth cranial nerve palsy [4-6].

## Case presentation

A 74-year-old woman with history of non-Hodgkin lymphoma, treated with six cycles of chemotherapy in March 2025 and in sustained remission [2], was admitted with progressive confusion, multiple falls, and a recent generalized tonic-clonic seizure. Her family reported a one-week history of persistent headache, dizziness, vomiting, and poor appetite. Four days before admission, she developed binocular diplopia, and clinical examination confirmed a left abducent nerve palsy [5,6]. This was initially thought to be traumatic in origin due to a minor fall, but subsequent imaging revealed no fractures or focal lesions.

Her past medical history included hypertension, asthma, and hypercholesterolemia. She was on aspirin for a recent stroke. She had no diabetes, no known chronic immunosuppressive condition, and no HIV risk factors. There was no significant family history of malignancy. She lived with family, was a non-smoker, and consumed alcohol only occasionally.

Upon admission, patient was afebrile, haemodynamically stable, and saturating well on room air. She was disoriented but able to follow basic commands. Cardiopulmonary and abdominal examinations were unremarkable. Neurological evaluation confirmed a left sixth nerve palsy without motor or sensory deficits [5]. Shortly after presentation, she had a self-limiting generalized seizure, managed with intravenous benzodiazepines.

**Figure 1:**
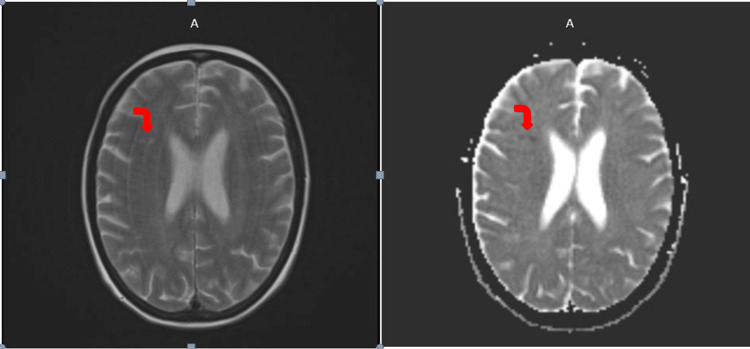
Axial MRI brain (T1- and T2-weighted sequences) showing right insular infarct (red arrows).

Laboratory investigations revealed chronic hyponatremia (124 mmol/L), mild hypokalemia (3.2 mmol/L), normal liver and renal function. A non-contrast CT of the head showed no acute haemorrhage or mass. Given her recent right insular infarct and worsening mental status, a contrast-enhanced MRI was obtained (Figure 1), which showed prior ischemic lesion with no evidence of new infarction, leptomeningeal enhancement, or malignancy [4]. 

**Figure 2:**
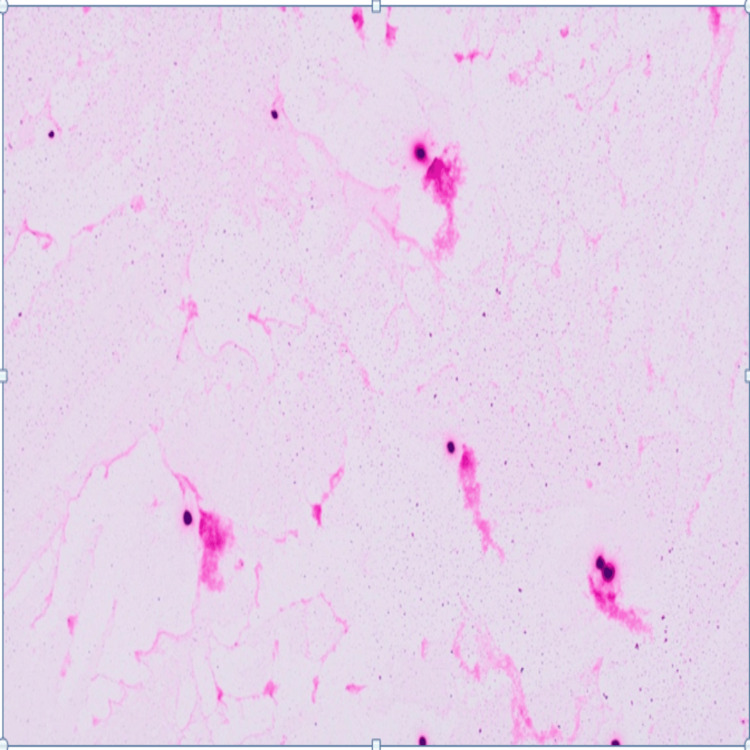
Gram stain showing budding yeast cells consistent with Cryptococcus neoformans.

Due to persistent confusion, a lumbar puncture was performed. The cerebrospinal fluid (CSF) was clear, with 32 white cells/µL (100% lymphocytes), protein 0.4 g/L, and glucose 0.6 mmol/L. Budding cryptococcal organism was seen in the Gram staining (Figure 2) and cryptococcal antigen testing was strongly positive with a titre 1:2650 along with polymerase chain reaction (PCR) confirmed *Cryptococcus neoformans* [1,7]. These findings established the diagnosis of cryptococcal meningitis. She was negative for HIV and other viral infections including cytomegalovirus (CMV), Epstein-Barr virus (EBV), hepatitis B and hepatitis C. Serial cerebrospinal fluid (CSF) parameters at diagnosis and after four weeks of treatment are summarized in Table 1 [7].

**Table 1:** Comparison of cerebrospinal-fluid parameters at baseline and after four weeks of antifungal therapy, demonstrating biochemical and microbiological improvement Abbreviations: LP: lumbar puncture; CSF: cerebrospinal fluid.

Parameter	Initial LP	Follow-up LP (after 4 weeks of therapy)	Interpretation
Appearance	Clear	Clear	-
White blood cell (WBC) count (/µL)	32 (100% lymphocytes)	39 (80% lymphocytes, 20% polymorphs)	Persistent mild lymphocytosis
Protein (g/L)	0.4	0.9	Rising trend reflecting immune recovery
Glucose (mmol/L)	0.6 (serum ≈ 5.7)	2.3 (serum ≈ 5.3)	Improving CSF:serum glucose ratio
Cryptococcal antigen titre	1:2650	1:320	Favourable serological response
Cryptococcal polymerase chain reaction (PCR)	Positive (Cryptococcus neoformans)	Negative	Microbiological clearance
Culture sensitivity and gram staining	Budding cryptococcal yeasts seen on gram stain	No growth seen	Further confirmation of infection clearance

Initial antifungal therapy included intravenous liposomal amphotericin B and high-dose oral fluconazole; flucytosine was added later when it became available [8]. Treatment was complicated by underlying cardiac dysfunction, necessitating cautious fluid management and close electrolyte monitoring to reduce the risk of amphotericin-related toxicity [8].

**Figure 3:**
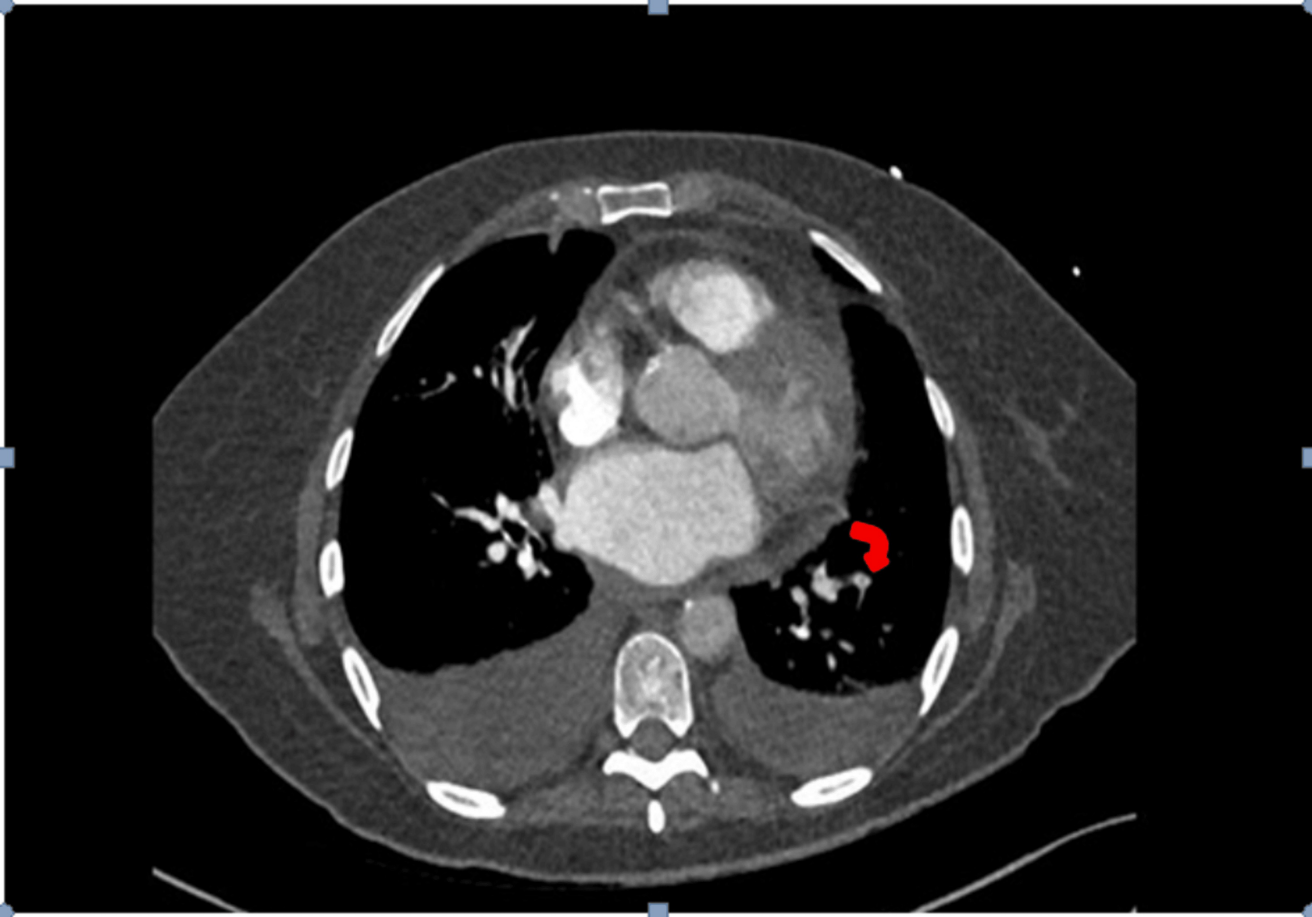
CT pulmonary angiogram showing segmental pulmonary emboli (red arrow)

During the second week of hospitalization, the patient developed acute dyspnoea and hypoxia, which was initially attributed to heart failure secondary to known anthracycline-induced cardiomyopathy [8]. She was treated with intravenous diuretics, resulting in a partial relief of symptoms. Echocardiography revealed concentric left-ventricular hypertrophy and severely reduced systolic function (ejection fraction 25%-30%). Due to persistent symptoms at the end of the second week, a CT pulmonary angiogram was performed, revealing a small sub-segmental pulmonary embolism as shown in Figure 3. Management included therapeutic anticoagulation, which was temporarily withheld during lumbar puncture procedures. This was later switched to apixaban under haematology guidance.

Neurology, infectious disease, cardiology, and neuroradiology teams collaboratively managed the patient [2,8]. The sixth-nerve palsy, initially attributed to trauma, improved progressively during antifungal therapy, supporting an inflammatory aetiology [5,6]. Repeat MRI after four weeks showed stable ventricles with no new infarcts or contrast enhancement [4]. Follow-up CSF examination demonstrated improved biochemical parameters, negative cryptococcal PCR, and reduced antigen titre, consistent with treatment response [7,8].

**Figure 4:**
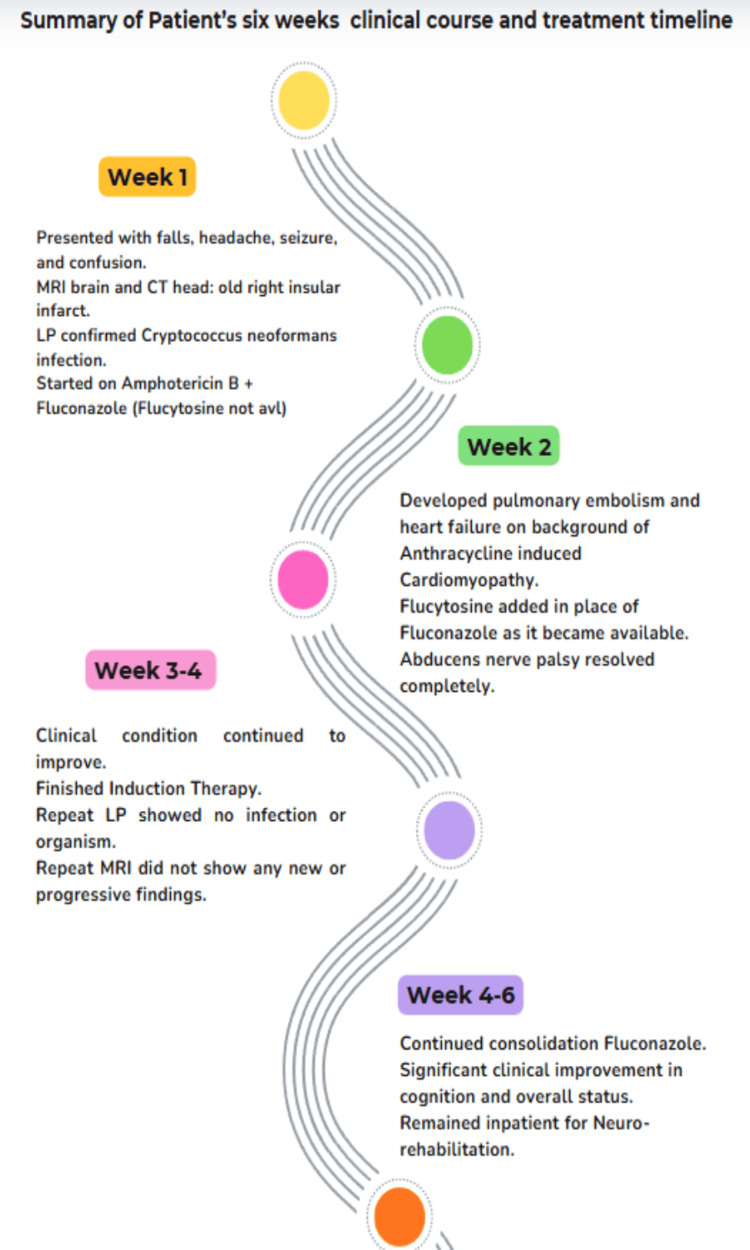
Timeline of events Image credit: Created using Canva by Dr. Sufiyan Jawed.

After four weeks of antifungal therapy, the patient showed steady neurological recovery. Cognitive function improved, seizures ceased and cranial-nerve palsy partially resolved. She remained haemodynamically stable, was tolerating oral nutrition and continued rehabilitation (physiotherapy and occupational therapy) under multidisciplinary care while receiving ongoing antifungal consolidation therapy. A summary of the timeline of events is shown in Figure 4.

## Discussion

Cryptococcal meningitis is a life-threatening fungal infection most commonly associated with severe immunosuppression [1,2]. While classically linked to advanced HIV infection, it is increasingly identified in patients with alternative forms of immunocompromise, including hematologic malignancies, organ transplants, or recent chemotherapy [1,2]. In HIV-negative individuals, diagnosis is often delayed due to lower clinical suspicion, contributing to increased morbidity.

This case highlights an unusual presentation of cryptococcal meningitis in an HIV-negative patient with previous chemotherapy [2]. The concurrent history of an insular infarct introduced diagnostic ambiguity [3]. Cerebral infarction may compromise local immune function and barrier integrity, facilitating CNS invasion by opportunistic pathogens [3]. This overlap in presentation between post-stroke encephalopathy and meningitis infection emphasizes the necessity of early CSF analysis in immunocompromised patients with new neurological symptoms.

The presence of a sixth-nerve palsy in this patient was initially thought to be post-traumatic but was later attributed to meningitic involvement [5,6]. Abducent nerve palsy is a recognized manifestation of cryptococcal meningitis, potentially resulting from increased intracranial pressure or direct nerve irritation [5]. This underscores the importance of integrating clinical findings with imaging and laboratory data rather than relying solely on presumed mechanisms.

Her clinical course was further complicated by cardiomyopathy and pulmonary embolism, both of which significantly impacted management [8]. Amphotericin B carries risks of nephrotoxicity and volume overload, which requires careful fluid management in the setting of reduced cardiac function [8]. The occurrence of a pulmonary embolism necessitated anticoagulation, creating additional complexity during serial lumbar punctures.

Treatment of cryptococcal meningitis involves induction with amphotericin B and flucytosine, followed by fluconazole for consolidation and maintenance [1,8]. In this case, flucytosine was not immediately available, delaying optimal dual therapy and increasing the length of initial phase. Nonetheless, once combination treatment commenced, the patient demonstrated biochemical and microbiological response with steady clinical improvement [7,8].

Hydrocephalus is a known complication in cryptococcal meningitis, particularly in HIV-negative patients [9]. In this case, early imaging showed no signs of meningeal enhancement. Serial neuroimaging was essential for monitoring disease progression and guiding enduring management [4,9].

This case offers several learning points. Opportunistic CNS infections like cryptococcal meningitis should be considered in immunocompromised, HIV-negative individuals with subacute neurological symptoms, especially when prior cerebrovascular injury may mask or mimic the presentation [3,10,11]. Multisystem comorbidities can complicate therapy and require a coordinated, multidisciplinary approach [2,8,12]. Most importantly, early lumbar puncture, appropriate antifungal initiation, and vigilant supportive care remain central to achieving favorable outcomes [1,8].

## Conclusions

We report a rare case of cryptococcal meningitis in an HIV-negative patient following chemotherapy for non-Hodgkin lymphoma and a recent insular infarct. The case was further complicated by cranial-nerve involvement, cardiomyopathy, and pulmonary embolism. This report underscores the need for maintaining a broad differential diagnosis in immunocompromised patients presenting with neurological decline. Early CSF evaluation, prompt antifungal treatment, and close coordination across specialties were crucial in achieving clinical and microbiological recovery. Individualized, multidisciplinary management remains essential when CNS infection occurs alongside systemic comorbidities.
